# Intensity-modulated radiotherapy for cervical esophageal squamous cell carcinoma without hypopharyngeal invasion: dose distribution and clinical outcome

**DOI:** 10.1093/jrr/rrz019

**Published:** 2019-05-13

**Authors:** Yuichi Ishida, Katsuyuki Sakanaka, Kota Fujii, Satoshi Itasaka, Takashi Mizowaki

**Affiliations:** Department of Radiation Oncology and Image-applied Therapy, Graduate School of Medicine, Kyoto University, 54 Sho-goin Kawahara-cho, Sakyo-ku, Kyoto, Japan

**Keywords:** cervical esophageal squamous cell carcinoma, radiotherapy, intensity-modulated, chemoradiotherapy, planning study

## Abstract

Hypopharyngeal invasion would be a key finding in determining the extent of the irradiation fields in patients with cervical esophageal squamous cell carcinoma (CESCC). This study aimed to investigate the clinical outcomes of chemoradiotherapy using simultaneous integrated boost intensity-modulated radiotherapy (SIB-IMRT) omitting upper cervical lymph nodal irradiation in CESCC without hypopharyngeal invasion, and the dosimetric superiority of SIB-IMRT to 3D conformal radiotherapy (3DCRT). We retrospectively identified 21 CESCC patients without hypopharyngeal invasion [clinical Stage I/II/III/IV (M1LYM); 3/6/5/7] (UICC-TNM 7th edition) who underwent chemoradiotherapy using SIB-IMRT between 2009 and 2015. SIB-IMRT delivered 60 Gy to each primary tumor and the metastatic lymph nodes, and 48 Gy to elective lymph nodal regions, including Levels III and IV of the neck, supraclavicular, and upper mediastinal lymphatic regions, in 30 fractions. The overall survival rate, locoregional control rate, and initial recurrence site were evaluated. 3DCRT plans were created to perform dosimetric comparisons with SIB-IMRT. At a median follow-up of 64.5 months, the 5-year locoregional control and overall survival rates were 66.7% and 53.4%, respectively. Disease progressed in eight patients: all were locoregional progressions and no patients developed distant progression including upper cervical lymph nodal regions as initial recurrence sites. The planning study showed SIB-IMRT improved target coverage without compromising the dose to the organs at risk, compared with 3DCRT. In conclusion, omitting the elective nodal irradiation of the upper cervical lymph nodes was probably reasonable for CESCC patients without hypopharyngeal invasion. Locoregional progression remained the major progression site in this population.

## INTRODUCTION

Chemoradiotherapy using the 3D conformal radiotherapy (3DCRT) technique yields a 29–66.5% 3-year overall survival (OS) rate in patients with cervical esophageal squamous cell carcinoma (CESCC) [1–5]. Two reports have suggested that this outcome is comparable with that for patients treated with surgery [6, 7]. However, locally advanced CESCC continues to show poor locoregional control (LRC) and OS [3–5]. One of the reasons for poor clinical outcome for locally advanced CESCC is the inadequate dose delivery to target organs using 3DCRT because of its technical limitations. Unlike 3DCRT, intensity-modulated radiotherapy (IMRT) is an advanced radiotherapy technique, which delivers conformal doses to the target but reduces doses to the spinal cord [8]. IMRT for CESCC has been reported as yielding better clinical outcome than that achieved with 3DCRT [9, 10].

The cervical esophagus is serially connected to the hypopharynx. Hypopharyngeal invasion of CESCC is not a rare situation and occurs in 11.1–42.6% of CESCC patients treated with chemoradiotherapy [5, 11–14]. The hypopharyngeal submucosa has abundant lymphatic drainage to the cervical lymph nodal regions. Once CESCC invades the hypopharynx, the risk of metastasis to the upper cervical lymph nodal regions (Level II and retropharyngeal lymph nodes) increases: 12% in CESCC patients with hypopharyngeal invasion vs 0–1% in CESCC patients without hypopharyngeal invasion, respectively [15–17]. Those pathophysiologic backgrounds suggest that the irradiation fields for the upper cervical lymph nodal regions are safely spared for CESCC without hypopharyngeal invasion. However, previous planning studies [8–10, 12–14, 18] did not consider the presence or absence of hypopharyngeal invasion in setting the extent of the irradiation fields, except for one study [11]. To our knowledge, no information is available on the clinical outcome of IMRT, and its dosimetric evaluation, for CESCC patients without hypopharyngeal invasion.

As of 2009, in our institution, CESCC patients without hypopharyngeal invasion underwent definitive chemoradiotherapy using simultaneous integrated boost intensity-modulated radiotherapy (SIB-IMRT). SIB-IMRT adopted elective nodal irradiation (ENI) omitting the upper cervical lymph nodal regions. In this study, we retrospectively reviewed the medical records of these patients and performed a planning study to compare SIB-IMRT with 3DCRT using ENI omitting the upper cervical lymph nodal regions. We aimed to examine the safety and effectiveness of chemoradiotherapy using SIB-IMRT to CESCC without hypopharyngeal invasion, omitting upper cervical lymph nodal irradiation, and carry out a dosimetric evaluation of SIB-IMRT.

## MATERIALS AND METHODS

### Inclusion criteria

This study retrospectively reviewed the medical records of patients who underwent SIB-IMRT for CESCC in our institution. All patients gave written informed consent for the research use of their clinical data prior to treatment. This study was reviewed and approved by the Institutional Review Board in our institution on 27 July 2016 (R0681) and on 16 March 2017 (R1048), and it was conducted in accordance with the Declaration of Helsinki and the Japanese Ethical Guidelines for Epidemiological Research.

From May 2009 to March 2015, 31 consecutive patients underwent definitive chemoradiotherapy using SIB-IMRT for CESCC in our institution. Among them, 21 patients met the following criteria: (i) histologically confirmed squamous cell carcinoma, (ii) the primary tumor was located in the cervical esophagus without hypopharyngeal invasion, (iii) there was a prescription of >50 Gy to the primary tumor and metastatic lymph nodes, and (iv) chemotherapy was concurrently administered. Ten patients were excluded from this study because of the following reasons: hypopharyngeal invasion (*n* = 8), non-use of chemotherapy (*n* = 1), and adenocarcinoma (*n* = 1).

This study collected the following information from the patients’ medical records: age, sex, Eastern Cooperative Oncology Group performance status, laboratory data, clinical stage of carcinoma (Union for International Cancer Control TNM Classification of Malignant Tumors, 7th edition), resectability, details of radiotherapy and chemotherapy, radiological findings [including computed tomography (CT) of the neck, chest and abdomen; magnetic resonance imaging of the neck and upper mediastinum; and ^18^F-fluorodeoxyglucose positron emission tomography (FDG-PET)], gastrointestinal endoscopic findings, and adverse events. Patient characteristics are listed in Table 1. Tracheal invasion or major vessel involvement of the primary tumor or metastatic lymph nodes was judged unresectable by the multidisciplinary oncology team (gastroenterologists, surgeons, medical oncologists, and radiation oncologists). ‘M1LYM’ indicates patients with supraclavicular and lower cervical lymph nodal metastasis that was included in the irradiation fields.

**Table 1.** Patient characteristics

Parameters	
Median age (IQR)	66 (57, 68)
Sex (female/male)	2/19
ECOG Performance Status (0/1/2)	16/5/0
Clinical stage (I/II/III/IV)^a^	3/6/5/7
T status (1/2/3/4)	5/5/2/9
N status (0/1/2/3)	5/13/3/0
M status (0/1LYM)	14/7
Location of M1LYM (supraclavicular/lower cervical/both)	5/1/1
Resectability (resectable/unresectable)	10/11

IQR = interquartile range, ECOG = Eastern Cooperative Oncology Group. M1LYM means patients with supraclavicular and lower cervical lymph nodal metastasis included in the irradiation fields. Unresectable status indicates patients with the tracheal invasion or major vessel involvement of the primary tumor or metastatic lymph nodes.

^a^Classification of clinical stage was based on the Union for International Cancer Control TNM Classification of Malignant Tumors, 7th edition.

### Details of delineation of SIB-IMRT

Delineation was performed in the included 21 patients as follows: CT simulation was performed with patients immobilized in the supine position. The primary tumor and metastatic lymph nodes were delineated based on physical examination, gastrointestinal endoscopy, and radiological findings. The clinical target volume (CTV) for the primary tumor (CTV_primary_) was created by adding a 0.5 cm margin in the radical direction and a 2.0 cm margin cranio-caudally of the primary tumor. The CTV for metastatic lymph nodes (CTV_node_) was created by adding a 0.5 cm margin in all directions from the metastatic lymph nodes. Those CTVs were manually modified to avoid overlap of bronchi, lungs and bones, which were considered anatomical barriers to tumor invasion. The planning target volumes (PTVs) for the primary tumor (PTV_primary_) and the metastatic lymph nodes (PTV_node_) were created by adding a 0.5 cm margin in all directions to the CTV_primary_ and the CTV_node_, respectively. The PTV_60_ was created from the sum of the PTV_primary_ and the PTV_node_. All patients underwent ENI. The CTV_ENI_ included the lymph node region from Level IIIs and IV of the neck, supraclavicular, and upper mediastinal lymphatic lesions to 1–3 cm caudally from the carina of the trachea. Then, the PTV_ENI_ was created by adding a 0.5 cm margin in all directions to the CTV_ENI_, excluding the volume overlapping with the PTV_60_. Moreover, the PTV_60_ and the PTV_ENI_ were cropped at 0.3 cm below the skin surface. The organs at risk (OARs) were delineated for the spinal cord, bilateral lungs, thyroid gland, and brachial plexus. The spinal cord plus a 0.3 cm margin formed the planning OAR volume (PRV_cord_). To evaluate the doses to the thyroid gland outside of the PTV_60_, we defined the thyroid_out of PTV_ as excluding the volume of overlap between the PTV_60_ and the thyroid gland. To evaluate the doses to the thyroid gland outside of the PTV60, we defined the thyroidout of PTV as excluding the volume of overlap between the PTV60 and the thyroid gland from the thyroid gland.

### Details of dose prescription and optimization of SIB-IMRT

Treatment plans were created using the SIB-IMRT method. The prescribed dose was 60 Gy to the PTV_60_ and 48 Gy to the PTV_ENI_ in 30 fractions. Optimizations were performed for prescribing a dose of 60 Gy as D_50%_ (D_*x*%_ represents the dose covering *x*% of the structure volume) of the PTV_60_ for eight patients from May 2009 to March 2011 and D_95%_ of the PTV_60_ for 13 patients from April 2011 to March 2015. Then, the included 21 patients underwent SIB-IMRT 5 days per week. The median overall treatment time was 42 days [interquartile range (IQR), 42–43]. The median total dose to the PTV_60_ and the PTV_ENI_ were 60 Gy (IQR, 60–60) and 48 Gy (IQR, 48–51), respectively.

SIB-IMRT plans used 4 or 6 MV photon beams. Seven to nine static coplanar fields were used in 19 patients from May 2009 to March 2014. We manually fixed the caudal jaw position in two lateral fields to the level of the top of the lungs to reduce the lung doses. From April 2014, volumetric-modulated arc therapy using coplanar two arcs was used for two patients. One arc rotated clockwise from 181° to 179°, and the other rotated counterclockwise from 179° to 181°. Avoidance sectors were introduced for bilateral 75° gantry angles for each arc to reduce lung doses. Avoidance sectors allowed the gantry to rotate with zero monitor unit output at preset angles (RapidArc system; Varian Medical Systems, Palo Alto, CA, USA).

We used dose constraints for the PTV_60_ as follows: D_2%_ <72 Gy, D_98%_ >55.8 Gy, and D_50%_ < 63 Gy; and for the PTV_ENI_ as follows: D_98%_ > 45.9 Gy, D_50%_ < 51 Gy, and D_2%_ < 60 Gy. The dose constraints for the OARs were also defined for the spinal cord and bilateral lungs as follows: PRV_cord_ for maximum point dose < 50 Gy, D_2 cm^3^_ (D_*x* cm^3^_ represents the dose covering *x* cm^3^ of the structure volume) < 46 Gy, and the lung V_10__Gy_ (V_*x* Gy_ represents the volume receiving *x* Gy) < 50%, V_15__Gy_ < 40%, and V_20__Gy_ < 25%. We did not set any dose constraints for the thyroid_out of PTV_ or the brachial plexus.

### Dose calculation of SIB-IMRT

Three types of dose calculation algorithms were used for the monitor unit settings, along with updates of the treatment planning systems and machine calibration as follows: pencil beam convolution version 8.2.23 (*n* = 2)/version 8.6.15 (*n* = 9), anisotropic analytical algorithm version 8.6.15 (*n* = 3)/version 11.0.31 (*n* = 2), and Acuros XB version 11.0.31 (*n* = 5). The dose distributions were calculated with a 2.5-mm grid size and included a heterogeneity correction (Batho Power Law method; Eclipse treatment planning system, Varian Medical Systems, Palo Alto, California, USA).

### Details of chemotherapy

Chemotherapy was administered twice every 4 weeks during radiotherapy. Nineteen patients received cisplatin (70 mg/m^2^ per day) by slow drip on Days 1 and 29, and 5-fluorouracil (700 mg/m^2^ per day) by continuous infusion for 24 h on Days1–4 and 29–32. One patient received only cisplatin (80 mg/m^2^ per day) by slow drip on Days 1 and 29 because of alcoholic cirrhosis, and another patient received only 5-fluorouracil (700 mg/m^2^ per day) by continuous infusion for 24 h on Days 1–4 and 29–32 because of advanced age.

### Follow-up

Initial evaluation of the tumor response was performed with endoscopy and CT 4–6 weeks after the last day of radiotherapy. Endoscopy was repeated every month until complete response or recurrence was confirmed. After complete response was confirmed, endoscopy and CT were repeated every 3–6 months. When it was difficult to judge recurrences with CT alone, FDG-PET was used. In addition to endoscopy and CT, physical examination and blood sampling were performed at each follow-up.

### End point of clinical outcome

LRC and OS rates were estimated with the Kaplan–Meier method. Survival was measured from the initial day of radiotherapy to the date of the last follow-up or death from any cause. We categorized the initial recurrence site into three types: locoregional progression, distant metastasis, and both. Locoregional progression was defined as progression of the primary tumor or metastatic lymph nodes and regional lymph nodal metastasis inside the irradiation fields. Metachronous superficial esophageal tumors outside the irradiation fields were not counted as locoregional progression events. Distant metastasis was defined as non-regional lymph nodal metastasis excluding supraclavicular lymph node or distant organ metastasis. Simultaneous development of locoregional progression and distant metastasis was categorized as both. The LRC rate was calculated from the initial day of radiotherapy to the day of locoregional progression. Adverse events were retrospectively evaluated using Common Terminology Criteria for Adverse Events, version 4.0. Adverse events within 90 days after the initial day of radiotherapy were defined as acute adverse events, and those occurring 91 days or more after initial day of radiotherapy were defined as late adverse events.

### 3DCRT planning

We created 3DCRT plans for all patients in order to compare the dose–volume indices of the 3DCRT plans and the SIB-IMRT plans that were used clinically [19]. The 3DCRT plans were created using the cone-down method and the same target volume used in the SIB-IMRT plans, by two board-certified radiation oncologists in the author group: one created all the 3DCRT plans, and the other one confirmed them. Neither of these radiation oncologists were involved in the SIB-IMRT planning. To avoid bias, they did not refer to the SIB-IMRT plans during 3DCRT planning. The 3DCRT planning was completed before starting the analysis of the SIB-IMRT plans. Initially, anterior–posterior plus parallel oblique radiation fields were used up to a total dose of 40 Gy, with 2.0 Gy fractions per day, which covered the PTV_60_ and the PTV_ENI_ and a 0.5 cm leaf margin in all directions. Boost irradiation was applied to the PTV_60_ using three to four coplanar fields, and the dose of the boost irradiation was 20 Gy, at a 2.0 Gy fraction per day. Although the leaf margin was set to 0.5 cm in all directions, reducing the leaf margin in the direction of the spinal cord was often necessary to reduce the dose to the spinal cord. Doses were prescribed to the isocenter in both the initial and boost plans.

### Dosimetric comparison between SIB-IMRT and 3DCRT

We used two types of dose prescription. We categorized the patients who underwent SIB-IMRT into two groups: those with a prescribed dose of 60 Gy delivered to 50% of the PTV_60_ (SIB-IMRT-D_50%_), and those with a prescribed dose of 60 Gy delivered to 95% of the PTV_60_ (SIB-IMRT-D_95%_). To compare the coverage of the gross tumor and OARs between two types of SIB-IMRT and 3DCRT, we created the dose–volume histograms of the PTV_60_, bilateral lungs, PRV_cord_, thyroid_out of PTV_ and brachial plexus. Then, we calculated the D_98%_, D_50%_, D_2%_, homogeneity index (HI) and conformation number (CN) of the PTV_60_, V_10__Gy_, V_15__Gy_ and V_20__Gy_ of the bilateral lungs; the maximum dose and D_2 cm^3^_ of the PRV_cord_, V_50__Gy_ and V_60__Gy_; the mean dose of the thyroid_out of PTV_, V_60__Gy_ and D_2 cm^3^_ of the brachial plexus; and the maximum dose and D_2 cm^3^_ of the V_out of PTV_, using each method. The HI was obtained using the following formula: HI = (D_2%_ – D_98%_)/D_50%_ [20]. The HI value should be more than 0 and as close to 0 as possible. The CN was defined as CN = (TV_RI_/TV) × (TV_RI_/V_RI_) [21]. TV_RI_, TV and V_RI_ represent the volume of the PTV receiving 60 Gy, the volume of the PTV, and the total volume receiving 60 Gy, respectively. The maximum value for the CN is 1: the closer the CN to 1, the better the conformity.

### Statistical analysis

Differences in OS and LRC rates between the categorized variable [age (≤66 vs >66), sex (male vs female), performance status (0 vs 1), T status (T1–3 vs T4), N status (N0 vs N1–2), M status (M0 vs M1LYM), resectability (resectable vs unresectable), and the types of the dose prescription (SIB-IMRT-D_95%_ vs SIB-IMRT-D_50%_)] were analyzed using the log-rank test. To evaluate the dosimetric differences between the two types of SIB-IMRT and 3DCRT, we used the Kruskal–Wallis test to compare the dose–volume indices of the PTV_60_, bilateral lungs, PRV_cord_, thyroid_out of PTV_, brachial plexus, and V_out of PTV_. All statistical tests were two-sided and carried out using EZR version 1.36 (Saitama Medical Center, Jichi Medical University, Saitama, Japan) [22], which is a graphical user interface for R (R Foundation for Statistical Computing, Vienna, Austria, version 3.0.2), and a *P*-value of <0.05 was considered to indicate statistical significance. More precisely, EZR is a modified version of R commander used to facilitate biostatistics.

## RESULTS

### OS, LRC, and initial recurrence sites

**Fig. 1.**
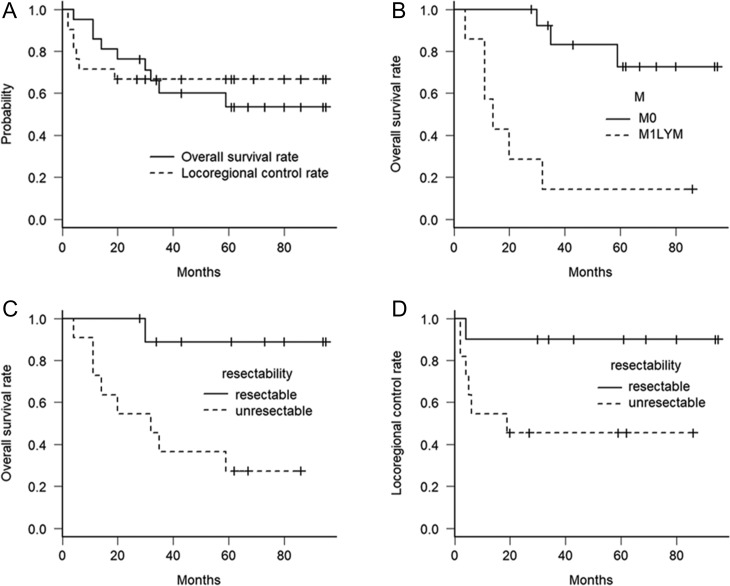
Kaplan–Meier curves for overall survival and locoregional control rate for all included patients (A), overall survival rate according to M status (B), and overall survival and locoregional control rate according to resectability (C, D). Abbreviations: M0 = patients without non-regional lymph nodal metastasis, M1LYM = patients with supraclavicular lymph nodes included in the irradiation fields, Unresectable = patients with tracheal invasion or major vessel involvement of the primary tumor or metastatic lymph nodes.

The median follow-up for all patients was 35 (range, 4–95) months, and the median follow-up for censored patients was 64.5 (range, 28–95) months. The 3- and 5-year OS rates were 60.0% and 53.4%, respectively, and the 3- and 5-year LRC rates were 66.7% and 66.7%, respectively (Fig. 1A).

Nine patients were dead at the time of analysis due to esophageal cancer progression (*n* = 6), other malignant tumors (*n* = 2), and sudden death (*n* = 1). Disease progressed in eight patients, and all initial recurrence sites were locoregional progression. That is, seven locoregional progressions occurred in the tumor beds of the the primary tumor or the metastatic lymph nodes, and the other was in the region of the ENI. Distant progression including upper cervical lymph nodal metastasis was not observed as the initial recurrence site. Metachronous superficial esophageal tumors were detected in three patients at 31, 47 and 52 months after the initial day of radiotherapy. All metachronous superficial esophageal tumors were outside the irradiation fields and completely resected by endoscopic treatment.

### Univariate analysis

M1LYM and unresectable statuses were associated with poor OS rate (*P* = <0.001 and 0.009). The 5-year OS rate was 72.7% for patients with M0 status vs 14.3% for patients with M1LYM status (Fig. 1B), and 88.9% for patients with resectable status vs 27.3% for patients with unresectable status (Fig. 1C). Other factors not associated with OS were age (*P* = 0.85), sex (*P* = 0.29), PS (*P* = 0.31), T status (*P* = 0.14), N status (*P* = 0.25), and the types of the dose prescription (*P* = 0.36).

Unresectable status was associated with a poorer LRC rate (*P* = 0.004) (Fig. 1D). The 5-year LRC rate was 90.0% for patients with resectable status vs 45.5% for patients with unresectable status. Other factors not associated with LRC were age (*P* = 0.35), sex (*P* = 0.34), PS (*P* = 0.14), T status (*P* = 0.44), N status (*P* = 0.095), M1LYM status (*P* = 0.053), and the types of the dose prescription (*P* = 0.11).

### Adverse events

Grade 3 or higher acute hematological toxicity occurred in five patients (5 leukopenia, 2 neutropenia and 1 anemia), and Grade 4 acute hematological toxicity occurred in 1 patient (leukopenia and neutropenia). Grade 3 acute non-hematological toxicity occurred in four patients (3 esophagitis, 1 anorexia, 1 esophageal stricture, 1 hyponatremia, 1 decreased renal function and 1 ischemic colitis). One patient developed a trachea–esophagus–lymph nodal fistula due to locoregional progression.

No patient developed late esophageal fistula or pleural effusion without recurrences. Grade 3 drug-induced interstitial lung disease was observed in one patient 8 months after the initial day of CRT, and Grade 3 esophageal stricture without recurrence was observed in two patients. Grade 3 or higher radiation pneumonitis was not observed. Grade 2 hypothyroidism was observed in 10 patients.

### Dosimetric comparison between 3DCRT and IMRT

**Fig. 2.**
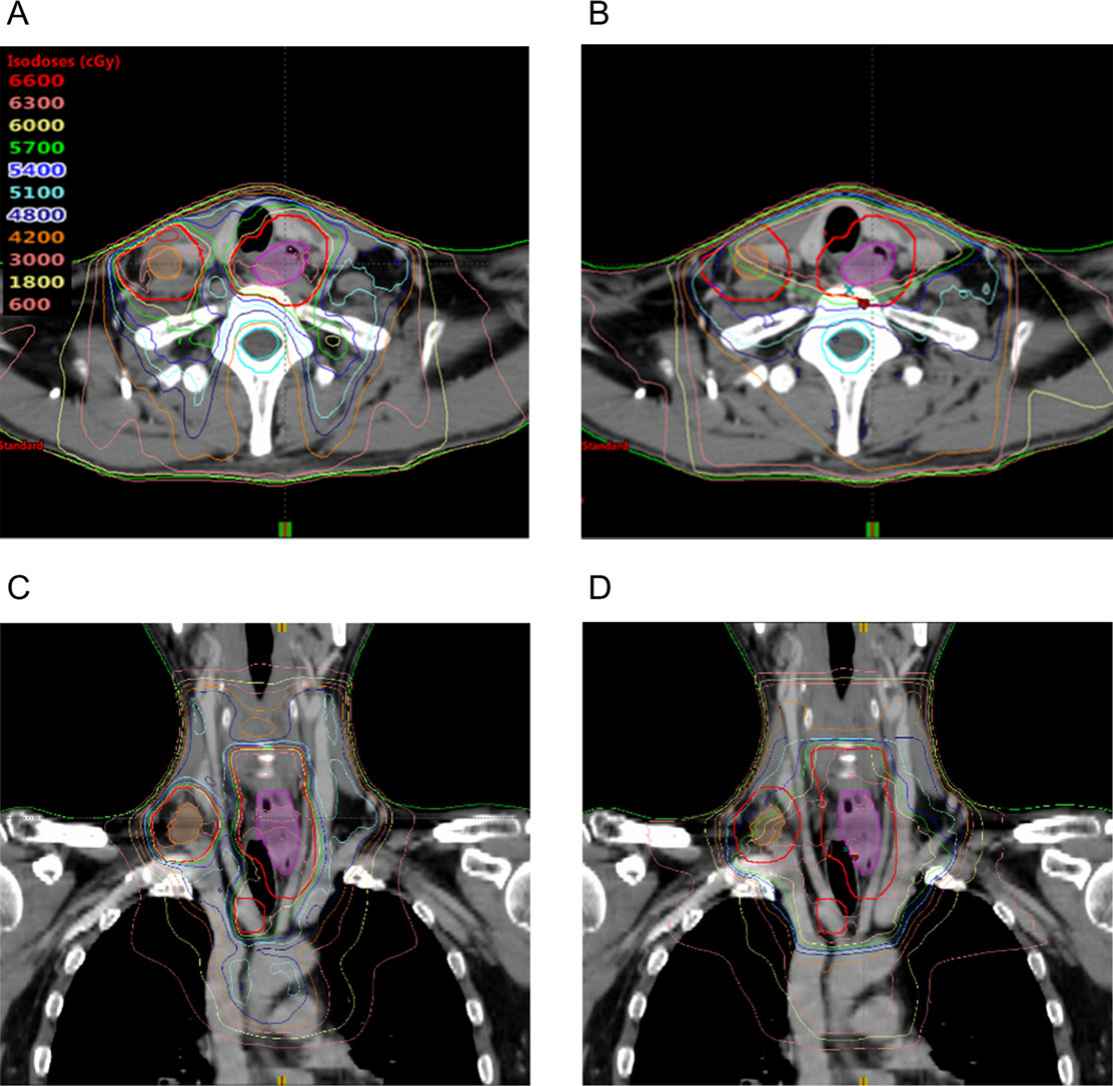
Axial and coronal images of the dose distribution of (A, C) simultaneous integrated boost intensity-modulated radiotherapy and (B, D) 3D conformal radiotherapy. Magenta translucent contour: primary tumor; orange translucent contour: metastatic lymph nodes; red contour: sum of the planning target volume of the primary tumor and the metastatic lymph nodes.

**Fig. 3.**
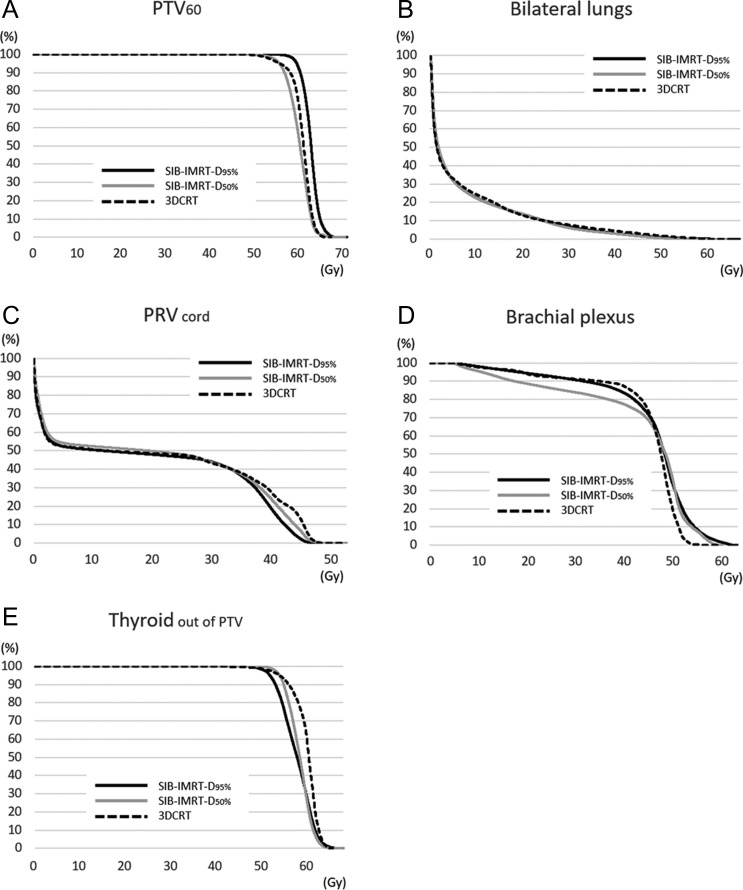
Mean cumulative dose–volume histograms of all patients for PTV_60_ (A), bilateral lungs (B), PRV_cord_ (C), brachial plexus (D), and Thyroid_out of PTV_ (E). Abbreviations: 3DCRT = 3D conformal radiotherapy, PTV_60_ = sum of planning target volume of primary tumor and metastatic lymph nodes, PRV_cord_ = the planning organs-at-risk volume of spinal cord, SIB-IMRT = simultaneous integrated boost intensity-modulated radiotherapy, SIB-IMRT-D_50%_: SIB-IMRT with the prescribed dose of 60 Gy delivered to 50% of PTV_60_, SIB-IMRT-D_95%_: SIB-IMRT with the prescribed dose of 60 Gy delivered to 95% of PTV_60_, Thyroid_out of PTV_ = volume of thyroid gland excluding the volume overlapping with PTV_60_.

Figure 2 shows the representative dose distribution in SIB-IMRT (Fig. 2A and C) and 3DCRT (Fig. 2B and D), and the mean dose–volume histograms of all included patients and dose–volume indices of the PTV_60_ and the OARs for each method are shown in Fig. 3 and Table 2, respectively. All dose–volume indices of the PTV_60_ differed significantly between the three methods (*P* < 0.001), and the *post hoc* analysis showed that SIB-IMRT-D_95%_ provided significantly better target coverage and target conformity of the PTV_60_ compared with 3DCRT (D_98%_, *P* < 0.001; D_50%_, *P* < 0.001; D_2%_, *P* < 0.001; HI, *P* = 0.0048; and CN, *P* < 0.001) and SIB-IMRT-D_50%_ (D_98%_, *P* < 0.001; D_50%_, *P* < 0.001; D_2%_, *P* = 0.018; HI, *P* < 0.001; and CN, *P* = 0.0015). In addition, there were no significant differences in the doses to the bilateral lungs and V_out of PTV_ between the two types of SIB-IMRT and 3DCRT. The PRV_cord_ and brachial plexus were safely spared by both types of SIB-IMRT and 3DCRT. Both types of SIB-IMRT significantly reduced the dose to the thyroid compared with 3DCRT.

**Table 2.** Comparison of the dose–volume indices of PTV_60_ and OARs between SIB-IMRT and 3DCRT

Dose index	3DCRT	SIB-IMRT-D_95%_	SIB-IMRT-D_50%_	*P*-value
PTV_60_				
Volume (ml) (IQR)	138.8 (96.8, 188.1)			
D_98%_ (Gy) (IQR)	54.3 (51.9, 56.1)	58.9 (57.8, 59.3)	53.7 (53.3, 54.0)	<0.001
D_50%_ (Gy) (IQR)	60.4 (60.3, 61.0)	62.4 (61.9, 63.0)	60.1 (59.7, 60.3)	<0.001
D_2%_ (Gy) (IQR)	63.5 (62.9, 63.7)	64.8 (64.5, 65.8)	63.1 (62.9, 63.7)	<0.001
HI (IQR)	0.135 (0.123, 0.182)	0.115 (0.086, 0.126)	0.155(0.150, 0.165)	<0.001
CN (IQR)	0.34 (0.32, 0.39)	0.77 (0.72, 0.79)	0.42 (0.36, 0.45)	<0.001
Bilateral lungs				
Volume (ml) (IQR)	2866 (2591, 3366)			
V_10__Gy_ (%) (IQR)	23.3 (21.5, 26.3)	22.2 (20.2, 24.2)	21.3 (19.7, 24.2)	0.39
V_15__Gy_ (%) (IQR)	17.7 (16.1, 21.7)	17.4 (15.5, 19.3)	16.9 (15.5, 18.2)	0.79
V_20__Gy_ (%) (IQR)	12.6 (11.4, 15.3)	13.7 (12.3, 15.4)	13.4 (12.5, 14.4)	0.51
PRV_cord_				
Volume (ml) (IQR)	137.2 (127.1, 148.5)			
Maximum dose (Gy) (IQR)	49.5 (48.8, 49.9)	48.8 (48.4, 49.2)	49.2 (47.3, 51.2)	0.22
D_2 cm^3^_ (Gy) (IQR)	46.6 (46.3, 47.2)	44.4 (43.9, 45.4)	45.2 (44.1, 46.8)	<0.001
Thyroid_out of PTV_				
Volume (ml) (IQR)	6.6 (3.8, 7.7)			
V_50__Gy_ (%) (IQR)	100 (98.7, 100)	99.5 (98.5, 100)	100 (100, 100)	0.02
V_60__Gy_ (%) (IQR)	74.7 (48.3, 79.0)	27.5 (16.4, 33.2)	29.2 (15.9, 48.2)	0.001
Mean dose (Gy) (IQR)	60.5 (59.4, 61.0)	57.6 (56.3, 58.5)	58.4 (57.8,59.9)	0.007
Brachial plexus				
Volume (ml) (IQR)	6.6 (5.9, 8.6)			
V_60__Gy_ (ml) (IQR)	0 (0, 0)	0 (0, 0.05)	0 (0, 0)	Not applicable
D_2 cm^3^_ (Gy) (IQR)	49.4 (48.5, 50.4)	49.8 (48.8, 51.4)	51.3 (50.5, 52.7)	0.03
V_out of PTV_				
Maximum dose (IQR)	64.6 (63.6, 65.1)	65.6 (64.0, 66.6)	64.0 (63.3, 64.7)	0.20
D_2 cm^3^_ (IQR)	62.2 (61.8, 63.3)	62.6 (61.9, 63.3)	61.6 (61.1, 62.2)	0.25

3DCRT = 3D conformal radiotherapy, HI = homogeneity index [20], CN = conformity number [21], D_*x* cm^3^_ = the dose covering *x* cm^3^ of the structure volume, D_*x*%_ = the dose covering *x*% of the structure volume, IQR = interquartile range, OARs = organs at risk, PRV_cord_ = the planning organs-at-risk volume of spinal cord, PTV_60_ = the sum of the planning target volume of the primary tumor and the metastatic lymph nodes, SIB-IMRT = simultaneous integrated boost intensity-modulated radiotherapy, SIB-IMRT-D_50%_: SIB-IMRT with the prescribed dose of 60 Gy delivered to 50% of PTV_60_, SIB-IMRT-D_95%_: SIB-IMRT with the prescribed dose of 60 Gy delivered to 95% of PTV_60_, Thyroid_out of PTV_ = volume of the thyroid gland excluding the volume overlapping with the PTV_60_, V_out of PTV_ = volume of the body out of all planning target volumes, V_*x* Gy_ = the volume receiving *x* Gy.

## DISCUSSION

The present study focused on the clinical outcome and dosimetric evaluation of CESCC patients without hypopharyngeal invasion. Locoregional progression remained the major progression site. No upper cervical lymph nodal metastasis occurred when omitting ENI for the upper cervical lymph nodal regions. The toxicity of SIB-IMRT was similar to that of 3DCRT. SIB-IMRT-D_95%_ for this population improved the target coverage and the conformity of the PTV without compromising the dose to the OARs, compared with 3DCRT.

Unresectable CESCC remained the negative prognostic factor for LRC in the present study. Unresectable status usually involves bulky or locally advanced esophageal carcinoma. Those factors are reported to be associated with poor OS and LRC in thoracic esophageal squamous cell carcinoma [23] and in CESCC patients treated with 3DCRT [3]. The present study suggested that SIB-IMRT-D_95%_ improved target coverage for CESCC. However, this was probably not enough to improve the clinical outcome for unresectable locally advanced CESCC. Zhao *et al.* reported that an intensified radiotherapy regimen, such as a total dose escalation to >66 Gy and a hypofractionated schedule using >2 Gy per fraction, improved LRC in CESCC [14]. Further development of the treatment strategy, such as dose escalation and concurrent chemotherapy regimens, are needed to improve LRC in unresectable CESCC patients.

Previous studies have reported that upper cervical lymph nodal metastasis occurs in 4.1–11.1% of patients after chemoradiotherapy for CESCC [4, 9, 24, 25]. Among these studies, one report suggested that the incidence of the upper cervical lymph nodal metastasis after chemoradiotherapy for CESCC depends on the existence of hypopharyngeal invasion: 4.2% without hypopharyngeal invasion vs 66.6% with hypopharyngeal invasion [25]. Our finding was consistent with that report. The present study showed that upper cervical lymph nodal metastasis was not observed as the initial recurrence site in CESCC patients without hypopharyngeal invasion when ENI was omitted for the upper cervical lymph nodal regions. Although further confirmatory study is necessary, omitting ENI for the upper cervical lymph nodal regions is probably reasonable for CESCC patients without hypopharyngeal invasion.

The incidence and severity of the adverse events in SIB-IMRT for CESCC were similar to those in 3DCRT. A comparison of these adverse events with those described in previous reports for 3DCRT are shown in Table 3. In previous reports, the most common Grade 3–4 acute hematological and non-hematological adverse events in CESCC patients treated with chemoradiotherapy using 3DCRT were leukopenia and esophagitis, respectively [1–4, 12, 25]. Further, late esophageal stricture occurred in 0–14.7% of patients in previous reports [1, 2, 4, 9, 12, 24]. Our result suggests that clinically adverse events when using SIB-IMRT was similar to those resulting when using 3DCRT. A further three reports compared the toxicity of 3DCRT with that of IMRT and showed there were no significant differences in the incidence and severity of the adverse events between them [9, 12, 18]. Our results indicated that SIB-IMRT for CESCC was a safe radiotherapy technique.

**Table 3.** Comparison of the adverse events with previous reports using 3D conformal radiotherapy

Author	*n*	Median prescribed dose (Gy)	Chemotherapy	≥ Grade 3 acute hematological (%)	≥ Grade 3 acute non-hematological (%)	≥ Grade 3 late esophageal stricture (%)
Zenda *et al.* [1]	30	60	5-FU/cisplatin	13.3	13.3 (mucositis)	13.3
Burnmeister *et al.* [2]	34	61.2	5-FU/cisplatin	11.8	14.7 (Grade 4)	14.7
Sakanaka *et al.* [3]	30	60	5-FU/cisplatin	3.3 (Grade 4)	13.3	0
Gkika *et al.* [4]	55	60	5-FU/cisplatin or cisplatin/etoposide	29 (leukopenia)	-	14.5
Ito *et al.* [9]	48	60	5-FU/cisplatin			8
Stuschke *et al.* [24]	17	60–65	cisplatin/etoposide	5.9 (Grade 4)	23.5	0
Yamada *et al.* [25]	27	66	5-FU/cisplatin	26	15	0
The current study	21	60	5-FU/cisplatin	23.8	19.0	9.5

The previous dosimetric planning study comparing IMRT with 3DCRT for CESCC had some flaws in the study design. First, the extent of ENI varied in each patient depending on the radiation oncologist [8]. Second, the dose–volume histogram of the lung was not correctly calculated in the previous report because the volume of the entire lung was not fully scanned. Contrary to a previous report, the present study adopted the same extent of ENI and scanned the whole volume of the lung in the dosimetric comparison between 3DCRT and IMRT. Based on this more appropriate study design, the present study showed the dosimetric superiority of SIB-IMRT-D_95%_ compared with 3DCRT in terms of target coverage and conformality, without increasing the doses to the OARs.

Nevertheless, the present study had limitations. This was a retrospective and single-institutional study. The number of included patients was small. The estimation of toxicity tended to include some bias. We did not set any dose constraints for the thyroid gland or the brachial plexus in the optimization of SIB-IMRT. The current study showed dosimetric advantages of SIB-IMRT-D_95%_ compared with 3DCRT for this population; however, further study is still needed to confirm whether SIB-IMRT improves the clinical outcome for CESCC patients. A multi-institutional Phase II study using SIB-IMRT for CESCC without hypopharyngeal invasion is ongoing (UMIN000009880), with a prescribed dose of 60 Gy delivered to 95% of the PTV_60_. This prospective study will yield important information on the effectiveness and safety of definitive chemoradiotherapy using SIB-IMRT for this population.

In conclusion, upper cervical lymph nodal metastasis was not observed, and ENI omitting the upper cervical lymph nodal regions might be a reasonable option for CESCC patients without hypopharyngeal invasion. Though SIB-IMRT-D_95%_ improved target coverage without increasing the dose to the OARs, compared with 3DCRT, locoregional area remained the major progression site in CESCC patients treated with SIB-IMRT.
